# MiR-106b-5p improving the progression of chronic kidney disease by inhibiting the TGF-β/Smad pathway

**DOI:** 10.1186/s41065-025-00468-7

**Published:** 2025-06-13

**Authors:** Qihao Ma, Li Xu, Peng Zhou, Bo Yang, Yong Li, Danan Sun

**Affiliations:** 1 Graduate School of Heilongjiang University of Traditional Chinese Medicine Harbin, Harbin, 150040 China; 2 Nephrology Department, The Second Affiliated Hospital of Heilongjiang, University of Chinese Medicine, No. 411, Guogeli Street, Nangang District, Harbin, 150001 Heilongjiang China

**Keywords:** CKD, miR-106b-5p, HK-2 cell, TGF-β/Smad

## Abstract

**Background:**

Chronic Kidney Disease (CKD) is a progressive disorder marked by renal impairment and declining kidney function.

**Aims:**

To investigate the expression of miR-106b-5p in CKD and its regulatory relationship with the TGF-β/Smad pathway.

**Methods:**

A total of 150 cases of CKD patients were selected as the observation group, while 100 healthy individuals served as the control group. Lipopolysaccharide (LPS) was utilized to induce damage to HK-2 cells. Real-time fluorescence PCR was used to detect the expression of genes. The CCK − 8 assay was utilized to evaluate cell proliferation, while flow cytometry was applied to measure the cell apoptosis rate.

**Results:**

miR-106b-5p is notably downregulated in CKD and exhibits a significant positive correlation with the eGFR in affected patients. Additionally, miR-106b-5p demonstrates a strong association with the levels of inflammatory factors in individuals with CKD. Furthermore, the expression of miR-106b-5p is reduced in LPS-induced HK-2 cells. Upregulation of miR-106b-5p can improve the inhibitory effect of LPS on the viability of HK-2 cells, reduce the apoptosis rate of cells, and alleviate the inflammatory response. miR-106b-5p serves as a negative regulatory factor within the TGF-β/Smad signaling pathway by directly targeting the pivotal receptor TGFBR2 and the downstream effectors SMAD2/3 within the TGF-β signaling cascade.

**Conclusions:**

miR-106b-5p ameliorates CKD progression by suppressing the TGF - β/Smad signaling pathway and could potentially be a therapeutic target for CKD.

**Supplementary Information:**

The online version contains supplementary material available at 10.1186/s41065-025-00468-7.

## Introduction

Chronic kidney disease (CKD) is a progressive disorder distinguished by renal damage and a decline in kidney function, characterized by high prevalence and significant mortality rates [1]. Its insidious onset often results in delayed diagnosis until advanced stages, where complications such as fibrosis and systemic inflammation become prominent [2]. In recent years, the incidence of CKD has been on the rise annually, establishing it as a crucial public health issue of global concern [3]. CKD is associated with a multitude of factors, including metabolic abnormalities, ischemia, hypoxia, infections, and autoimmune reactions [4, 5]. The persistent presence of these damaging factors leads to diminished renal microcirculation perfusion, progressive fibrosis, and an unavoidable progression towards end-stage renal disease [6]. As the disease advances, the risk of cardiovascular disease (CVD) and mortality among CKD patients correspondingly escalates, resulting in profound negative repercussions for both patients and their families [7]. Early detection of renal dysfunction and enhanced comprehensive management are therefore critical for effective diagnosis and treatment of CKD patients.

It has been firmly established that MicroRNAs (miRNAs) play a key role in controlling the pathological mechanisms of multiple diseases [8]. In particular, CKD has seen a growing body of evidence highlighting the diagnostic potential of circulating miRNAs, with an increasing focus in translational research on their applications in disease staging and prognostic evaluation [9]. Research indicates that miR-214 could act as both a therapeutic target and a diagnostic biomarker for CKD [10]. The investigations have indicated that miR-122-5p modulates renal fibrosis in vivo [11]. miR-29a-5p has been found to exhibit abnormal expression patterns in CKD [12]. Cohort studies show circulating miR-106b-5p correlates significantly with renal function parameters in coronary angiography patients [13]. Systematic reviews of miRNA profiles in CKD consistently show lower miR-106b-5p expression in CKD [14]. miR-106b-5p is involved in cell proliferation, apoptosis, and inflammation, but its role in CKD remains unclear. The TGF-β/Smad pathway plays a critical role in renal fibrosis. Thus, it is hypothesized that miR-106b-5p may intervene in CKD progression by targeting key nodes of the TGF-β/Smad pathway.

This study aims to investigate the expression of miR-106b-5p in CKD and its regulatory relationship with the TGF-β/Smad pathway, providing new theoretical insights and potential therapeutic targets for CKD prevention and treatment.

## Materials and methods

### Clinical data

A total of 150 patients diagnosed with chronic kidney disease, treated at The Second Affiliated Hospital of Heilongjiang, University of Chinese Medicine between January 2023 and June 2024, were selected for the observation group. Concurrently, 100 healthy volunteers undergoing routine physical examinations during the same period were designated as the control group. Inclusion criteria: Patients with a confirmed diagnosis who meet the staging and diagnostic criteria for CKD; those with complete clinical data. Exclusion criteria: Individuals with immunodeficiency; patients with nephrotic syndrome or acute renal failure; patients who have received peritoneal dialysis or hemodialysis within the past six months. All participants provided voluntary informed consent, and the study received approval from the hospital’s medical ethics committee.

The estimated glomerular filtration rate (eGFR) was determined utilizing the CDK-EPI formula, with patients categorized according to the 2012 KDIGO guidelines for assessing the risk of CKD progression [15]. In accordance with the eGFR (mL/min/1.73 m²) levels, CKD stages were delineated as follows: G1 (eGFR ≥ 90), G2 (eGFR 60–89), G3a (eGFR 45–59), G3b (eGFR 30–44), G4 (eGFR 15–29), and G5 (eGFR < 15) [16]. The clinical data of the subjects were collected. Fasting venous blood samples were obtained from all patients the day following admission (with the control group being sampled on the day of physical examination). Subsequently, the samples were allowed to reach room temperature naturally, and then serum was isolated.

### Cell cultivation and treatment

Human renal proximal tubular epithelial cells (HK-2) were obtained from Wuhan Purocell Life Science & Technology Co., Ltd. The HK-2 cells were sustained in DMEM/F12 complete medium with the addition of 10% fetal bovine serum and were cultured in a 5% CO_2_ humidified atmosphere at a temperature of 37 °C.

HK-2 cells in the logarithmic growth phase were digested, collected, centrifuged and resuspended. The cells were then counted and the density adjusted to 5 × 10⁴ cells/ml. Subsequently, 200 µL of the cell suspension (1 × 10⁴ cells per well) was seeded into 96-well plates, with three parallel wells set for each group. After the cells adhered to the wall for 24 h, the original culture medium was discarded and DMEM medium containing 10 µg/mL lipopolysaccharide (LPS) (with 2% FBS and 1% double antibiotics) was added [17].The LPS-containing medium was replaced every 24 h for a continuous 72-hour treatment to establish a chronic inflammation model.

The specific transfection operation steps were followed as per the Lipofectamine ^TM^ 2000 reagent user manual. The cells were randomly divided into the control group (Untreated), the LPS group, the LPS + mimic NC group (transfected with mimic negative control, 50 nmol/L), the LPS + miR-106b-5p mimic group (transfected with miR-106b-5p mimic, 50 nmol/L), the LPS + inhibitor NC group (transfected with inhibitor negative control, 50 nmol/L), and the LPS + miR-106b-5p inhibitor group (transfected with miR-106b-5p inhibitor, 50 nmol/L). After 48 h, HK-2 cells and supernatant from each group were collected for corresponding index detection.

### Real-time quantitative PCR

Total RNA was extracted using Trizol reagent (Invitrogen, USA) and its concentration. The Nanodrop spectrophotometer was used to detect the concentration and purity of the extracted total RNA. A 2µL RNA sample was added to the detection platform and its absorbance (A value) at 260 nm and 280 nm is measured. The A₂₆₀/A₂₈₀ ratio of the RNA samples within the range of 1.8–2.2 is used for the subsequent experiments. The SuperScript™ III Reverse Transcriptase Kit (Invitrogen, USA) is used for reverse transcription to synthesize cDNA. The levels of miR-106b-5p in serum and each group of HK-2 cells were detected by ABI 7500 RT-qPCR instrument (ABI, USA). Reaction procedure is as follows: pre-denaturation at 95 ℃ for 15 min; denaturation at 96 ℃ for 15 min, annealing at 57 ℃ for 30 s, extension at 72 ℃ for 30 s, and 40 cycles in total. GAPDH was used as an internal reference, and the level of miR-106b-5p was calculated by the 2^−ΔΔCt^ method (Ct is the threshold value).

### CCK-8 cell viability assay

Cell viability was assessed via the CCK-8 assay, which detects mitochondrial succinate dehydrogenase activity. This enzyme converts water-soluble WST-8 into a colored formazan product, with yield proportional to viable cell count. The method reflects metabolic activity: intact mitochondrial function in live cells enables color development, whereas dead cells show no signal due to metabolic arrest. HK-2 cells from each group were seeded into 96-well culture plates. Once the treatments for each group were finished, the CCK-8 detection reagent (Elabscience, Wuhan) was added to every well, and the plates were placed in the incubator for a 2-hour incubation period. Then, the absorbance (A) value of each well was measured at a wavelength of 450 nm to accurately reflect cell viability, distinguishing between live and non-live cells via metabolic activity. Proliferation Activity (%) = (A value of the experimental group / A value of the control group) × 100%.

### Apoptosis rate detection

The HK-2 cells in each group were collected and resuspended to achieve a cell density of 2 × 10^6^ cells/ml. Collect at least 5000 cells per group, and perform three biological replicates for each group. 100 µL of cell suspension was transferred to the flow cytometry tube, and 5 µL of Annexin V-FITC was added. The mixture was gently mixed and incubated at room temperature in the dark for 15 min. After the incubation, 5 µL of propidium iodide (PI) was added and gently mixed, and incubated at room temperature in the dark for 5 min. In the flow cytometry analysis software, forward scattering (FSC) and side scattering (SSC) were used to exclude cell debris and impurities. Based on the FSC and SSC characteristics of the cells, the target cell population was circled, and small or large fragments and irregularly shaped impurities were excluded. Then, in the fluorescence channels (FL1 detected the green fluorescence of vinculin V-FITC, FL3 detected the red fluorescence of propidium iodide), using unstained cells as a negative control, an appropriate fluorescence threshold was set to distinguish negative cells from positive cells. At least 5000 cells were collected from each group, and each group was subjected to 3 biological replicates. The BD Accuri™ C6 flow cytometer was used to evaluate the apoptosis rate of cells.

### Enzyme-linked immunosorbent (ELISA) assay

The concentrations of Interleukin-6 (IL-6), Tumor Necrosis Factor-α (TNF-α), Interleukin-1β (IL-1β), and Interleukin-10 (IL-10) in the serum of all subjects as well as in the supernatants of HK-2 cells from each group were evaluated through the ELISA technique. The process was carried out in strict accordance with the instructions provided by the test kit (Fine Test, Wuhan). Human TGF-β1 DuoSet ELISA (R&D Systems) was used to detect TGF-β1 in the serum and the supernatant of each group of cell cultures.

### Dual-luciferase reporter gene assay

HK-2 cells were plated in 48-well culture plates. They were then co-transfected with miR-106b-5p mimics and either the wild-type forms (TGFBR2-WT, SMAD2-WT, SMAD3-WT) or the mutant forms (TGFBR2-MUT, SMAD2-MUT, SMAD3-MUT) of the target genes, which were integrated into a dual-luciferase reporter system. Following a 48-hour incubation post-co-transfection, the luciferase activities of both the sea urchin and firefly were assessed. The relative luciferase activity for the cells was calculated as the ratio of the two luminescence readings.

### Statistical methods

The data were meticulously processed utilizing SPSS version 26.0 and GraphPad version 9.0 software. ANOVA was performed to allow for comparisons across several groups. If the data adhered to the required assumptions of normality and variance homogeneity, the Least Significant Difference (LSD) test was utilized. In the case of normally distributed data with unequal variances, the Games-Howell method was chosen. When the data did not conform to a normal distribution pattern, the non-parametric Kruskal-Wallis test was employed for the analysis. *P* < 0.05 was regarded as indicating statistical significance.

## Results

### Comparison of clinical data

A cohort of 150 patients with CKD was stratified into six subgroups based on estimated glomerular filtration rate (eGFR): G1 (*n* = 24), G2 (*n* = 27), G3a (*n* = 30), G3b (*n* = 34), G4 (*n* = 23), and G5 (*n* = 12). Baseline characteristics, including age, gender, body mass index (BMI), and prevalence of underlying diseases, demonstrated no statistically significant heterogeneity across the subgroups (*p*>0.05). In contrast, statistically significant inter-group differences were observed in the levels of total cholesterol (TC), triglycerides (TG), low-density lipoprotein cholesterol (LDL-C), and high-density lipoprotein cholesterol (HDL-C) (*p*<0.01) (Table 1).

**Table 1:** General information and biochemical indicators of the subjects

Variable	Control (*n* = 100)	CKD (*n* = 150)						*P* value
		G1	G2	G3a	G3b	G4	G5	
		(*n* = 24)	(*n* = 27)	(*n* = 30)	(*n* = 34)	(*n* = 23)	(*n* = 12)	
age (years)	53.65 ± 13.72	52.79 ± 12.76	54.11 ± 11.63	56.00 ± 11.18	56.94 ± 10.02	57.52 ± 10.06	62.08 ± 7.88	0.220
gender (male/female)	44/56	13/11	15/12	17/13	21/13	14/9	7/5	0.523
BMI (kg /m^2^)	25.72 ± 3.23	26.65 ± 3.02	26.15 ± 3.14	26.48 ± 2.72	27.23 ± 2.97	26.98 ± 2.61	27.12 ± 2.73	0.159
hypertension (yes/no)	40/60	14/10	14/13	15/15	21/13	15/8	8/4	0.125
diabetes (yes/no)	22/78	6/18	11/16	12/18	15/19	10/13	5/7	0.085
TC (mmol/L)	4.23 ± 0.35	5.54 ± 0.52	5.76 ± 0.65	5.38 ± 0.65	5.21 ± 0.63	5.10 ± 0.63	4.99 ± 0.58	<0.001***
TG (mmol/L)	1.16 ± 0.48	2.01 ± 0.19	1.98 ± 0.17	1.98 ± 0.16	2.07 ± 0.18	2.20 ± 0.42	2.09 ± 0.43	<0.001***
LDL-C(mmol/L)	2.72 ± 0.53	3.04 ± 0.48	2.94 ± 0.58	3.02 ± 0.51	3.05 ± 0.62	2.88 ± 0.50	3.03 ± 0.59	0.009**
HDL-C(mmol/L)	1.47 ± 0.19	1.10 ± 0.12	1.09 ± 0.11	1.13 ± 0.10	1.09 ± 0.13	1.07 ± 0.12	1.06 ± 0.10	<0.001***

BMI, Body mass index; TC, Serum total cholesterol; TG, Triglycerides; HDL-C, High-density lipoprotein cholesterol; LDL-C, Low-density lipoprotein cholesterol. **P < 0.01, ***P < 0.001

### Expression of miR-106b-5p and its correlation with eGFR

**Fig. 1:**
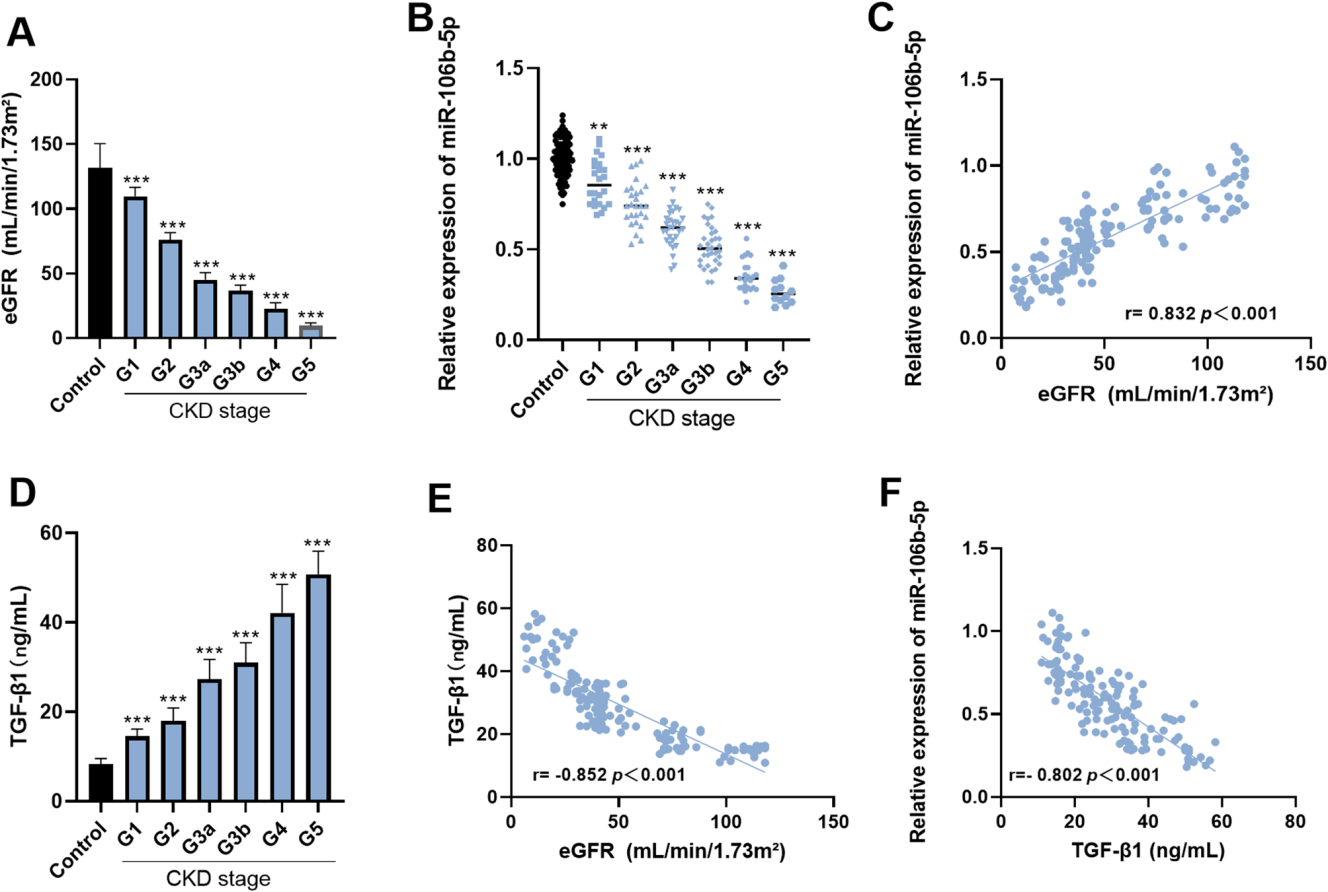
Analysis of the expression level of miR-106b-5p and TGF-β1 in CKD patients. The eGFR of CKD patients was significantly lower, reaching its lowest level in the G4-G5 stage, compared to the control group (*p* < 0.001) (**A**). The expression of serum miR-106b-5p in CKD patients decreases with increasing CKD stage and reaches its lowest level in stages G4-G5 (*p* < 0.001) (**B**). miR-106b-5p is positively correlated with the level of eGFR in patients with CKD (*r* = 0.832, *p* < 0.001) (**C**).The serum TGF-β1 levels in patients with CKD in the G1-G5 stages showed a gradually increasing trend (*p* < 0.001) (**D**). The TGF-β1 level in CKD patients is significantly negatively correlated with the eGFR level (*r* = 0.852, *p* < 0.001) (**E**). Additionally, the TGF-β1 level is negatively correlated with the expression level of miR-106b-5p (*r* = 0.802, *p* < 0.001) (**F**). ** *p* < 0.01, *** *p* < 0.001

Relative to the control subjects, eGFR levels in CKD patients were significantly lower. With the advancement of CKD stages, these levels showed a progressive decline, reaching their nadir in the G4–G5 stages (*p* < 0.001) (Fig. 1A). According to the results of RT-qPCR analysis, the level of serum miR-106b-5p expressed in CKD patients (stages G1–G5) was remarkably lower when contrasted with the controls (*p* < 0.05). Furthermore, miR-106b-5p levels exhibited a stage-dependent decline (*p* < 0.001) (Fig. 1B). According to the results of the Pearson correlation analysis, a strong positive association was found between the serum miR-106b-5p levels and the eGFR levels in individuals with CKD (*r* = 0.832, *p* < 0.001) (Fig. 1C).

### Analysis of TGF-β1 levels in CKD patients

The ELISA results showed that, compared with the control group, the serum TGF-β1 levels in patients with CKD in the G1-G5 stages showed a gradually increasing trend (*p* < 0.001) (Fig. 1D). The relevant analysis results show that the serum TGF-β1 level in CKD patients is significantly negatively correlated with the eGFR level (*r*= -0.852, *p* < 0.001) (Fig. 1E). Additionally, the TGF-β1 level is negatively correlated with the expression level of miR-106b-5p (*r*= -0.802, *p* < 0.001) (Fig. 1F).

### Correlation between miR-106b-5p and inflammatory factor levels in CKD patients

**Fig. 2:**
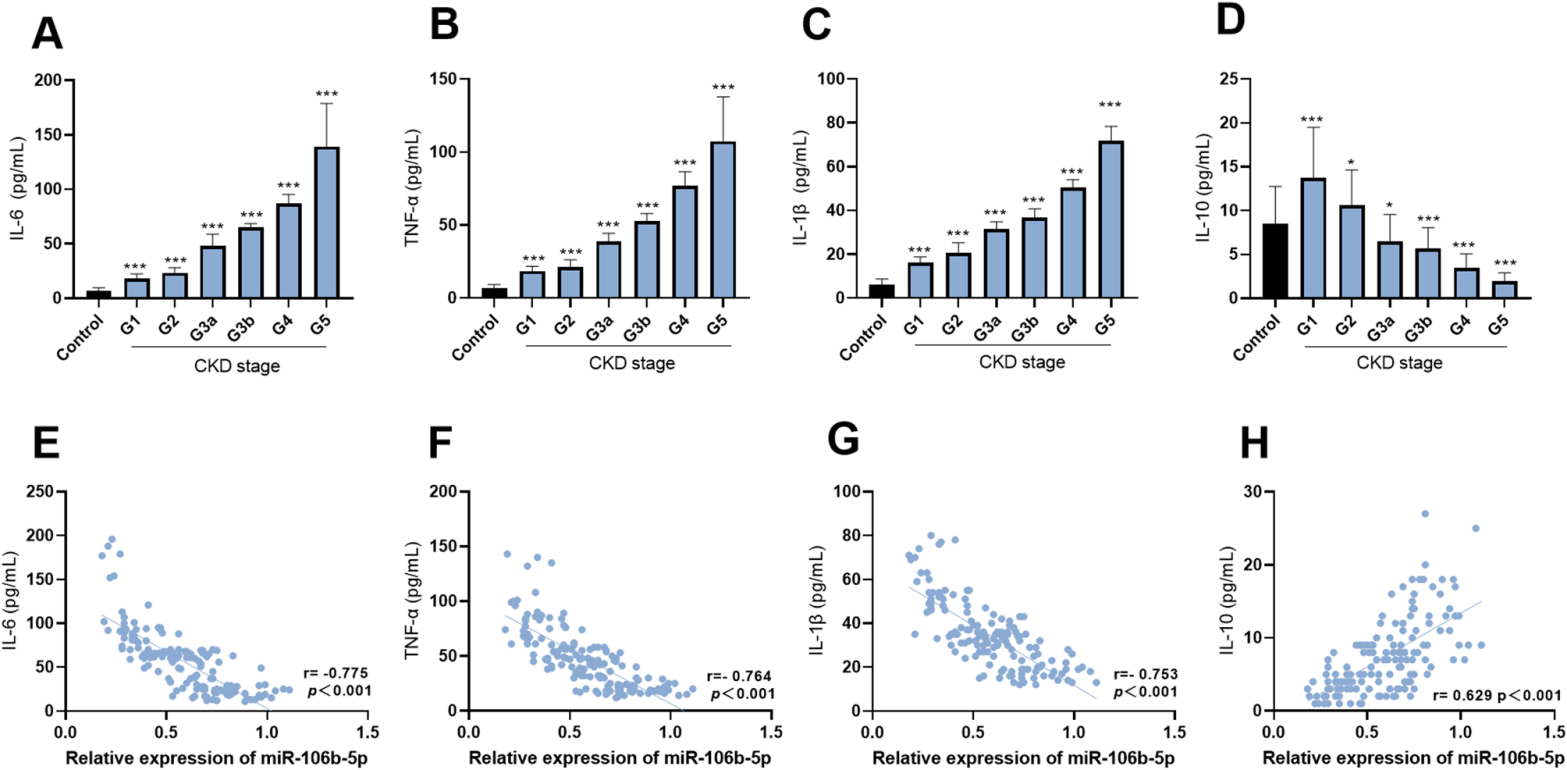
Correlation Between miR-106b-5p and Inflammatory Factor Levels in CKD Patients. As CKD progresses, there is a significant increase in the levels of IL-6, TNF-α and IL-1β *(p* < 0.001) (**A-C**). The level of IL-10 increased in patients during the G1-G2 stage, but decreased during the G3-G5 stage (*p* < 0.05) (**D**). The Pearson correlation analysis revealed that in CKD patients, miR-106b-5p was significantly negatively correlated with inflammatory factors IL-6, TNF-α and IL-1β (**E-G**), while it was significantly positively correlated with the anti-inflammatory factor IL-10 (**H**). * *p* < 0.05, *** *p* < 0.001

ELISA analysis showed that, relative to the control group, the levels of IL − 6, TNF - α, and IL − 1β increased significantly as CKD progressed (*p* < 0.001) (Fig. 2A-C). The level of IL-10 increased in patients during the G1-G2 stage, but decreased during the G3-G5 stage (*p* < 0.05) (Fig. 2D). The Pearson correlation analysis revealed that in CKD patients, miR-106b-5p was significantly negatively correlated with inflammatory factors IL-6 (*r*= -0.775), TNF-α (*r*= -0.764), and IL-1β (*r*= -0.753) (*p* < 0.001) (Fig. 2E-G), while it was significantly positively correlated with the anti-inflammatory factor IL-10 (*r* = 0.629, *p* < 0.001) (Fig. 2H).

### Effect of miR-106b-5p on LPS-induced HK-2 cells

**Fig. 3:**
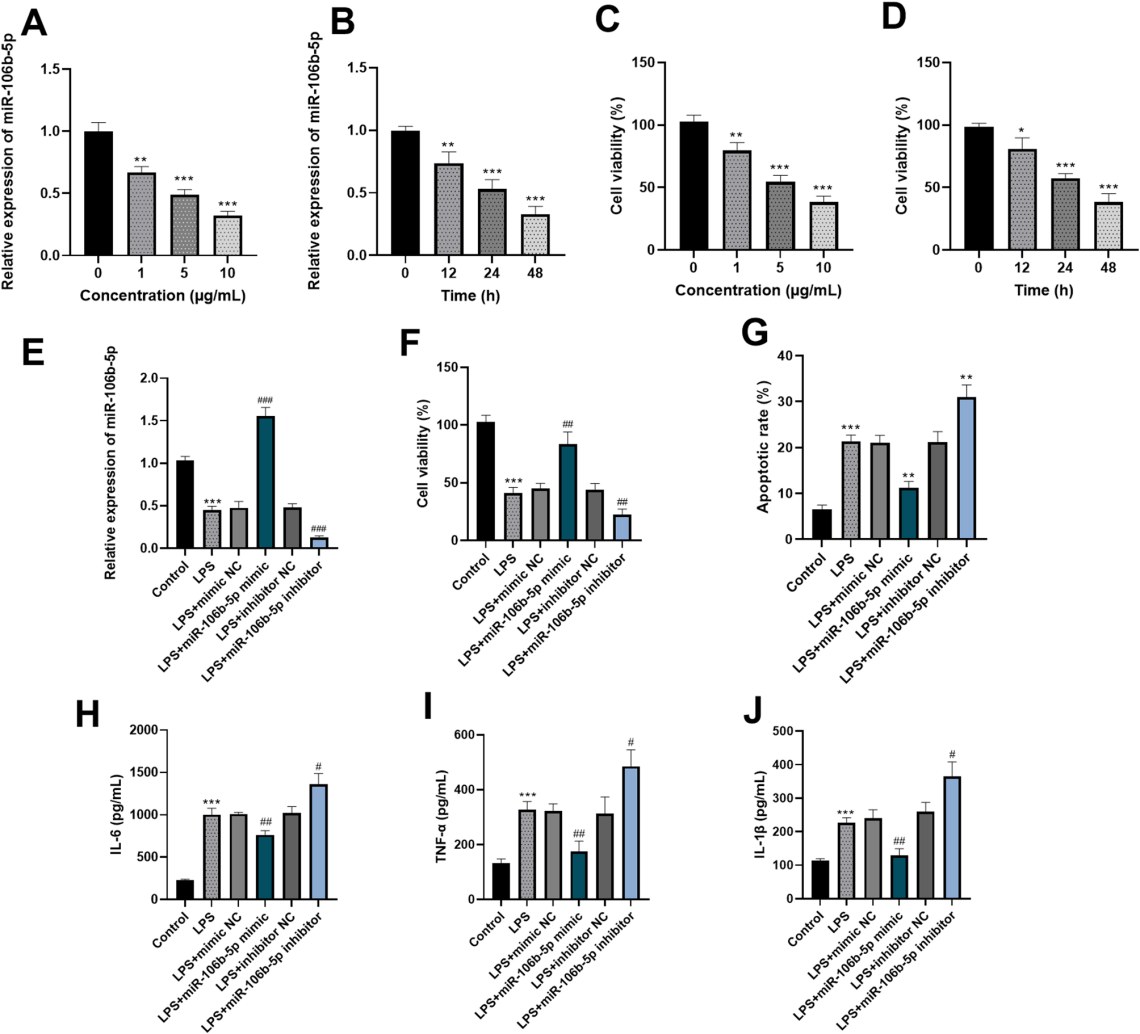
Effect of miR-106b-5p on LPS-induced HK-2 cells. RT-qPCR was used to determine the expression of miR-106b-5p in LPS-induced HK-2 cells (**A-B**). The CCK-8 assay was used to determine the effect of LPS on the viability of HK-2 cells (**C-D**). The expression of miR-106b-5p was determined by RT-qPCR as a confirmation of the transfection efficiency of the cells (**E**). miR-106b-5p can ameliorate the inhibitory effect of LPS on the viability of HK-2 cells, reduce the apoptosis rate of cells (**F-G**). miR-106b-5p can alleviate the pro-inflammatory effect of LPS on HK-2 cells (**H-J**). * *p* < 0.05, ** *p* < 0.01, *** *p* < 0.001

The results of RT-q PCR showed LPS treatment downregulated miR-106b-5p expression in HK-2 cells compared to the control group, and its level decreased with the increase of LPS concentration (*p*<0.05) (Fig. 3A). Furthermore, as the induction time increased, the level of miR-106b-5p showed a downward trend (*p*<0.05) (Fig. 3B). The results from the CCK-8 cell viability assay demonstrated that HK-2 cells stimulated by LPS had reduced viability relative to the control group. Additionally, with an increase in the LPS concentration, the cell viability steadily decreased (*p*<0.01) (Fig. 3C). Additionally, the viability of HK-2 cells was found to decrease in conjunction with an extended induction time in the presence of LPS (*p*<0.05) (Fig. 3D).

To investigate the impact of miR-106b-5p on LPS-induced HK-2 cells, we employed cell transfection techniques to achieve both overexpression and knockdown of miR-106b-5p. As depicted in Fig. 3E, in contrast to the control group, there was a substantial rise in the miR-106b-5p expression within the LPS + miR-106b-5p mimic group. Meanwhile, the miR-106b-5p expression in the LPS + miR-106b-5p inhibitor group was markedly decreased (*p* < 0.001). As evidenced by the CCK-8 cell viability assay results, LPS impaired the viability of HK-2 cells. However, the elevated expression of miR-106b-5p efficiently offset this negative effect. In contrast, the knockdown of miR-106b-5p intensified LPS-induced cell death (*p*<0.01) (Fig. 3F). Additionally, the assessment of the cell apoptosis rate revealed that LPS promoted apoptosis in cells. Notably, miR-106b-5p overexpression inhibited apoptosis, while its knockdown exacerbated it, indicating that miR-106b-5p plays a protective role against LPS-induced apoptosis (*p* < 0.01) (Fig. 3G). Notably, ELISA analysis of HK-2 cell supernatants showed LPS significantly increased pro-inflammatory cytokines IL-6, TNF-α, and IL-1β. Notably, miR-106b-5p upregulation reduced these cytokine levels, while its knockdown promoted inflammatory factor release (*p* < 0.05) (Fig. 3H-J).

### miR-106b − 5p targets and inhibits the TGF-β/SMAD pathway

**Fig. 4:**
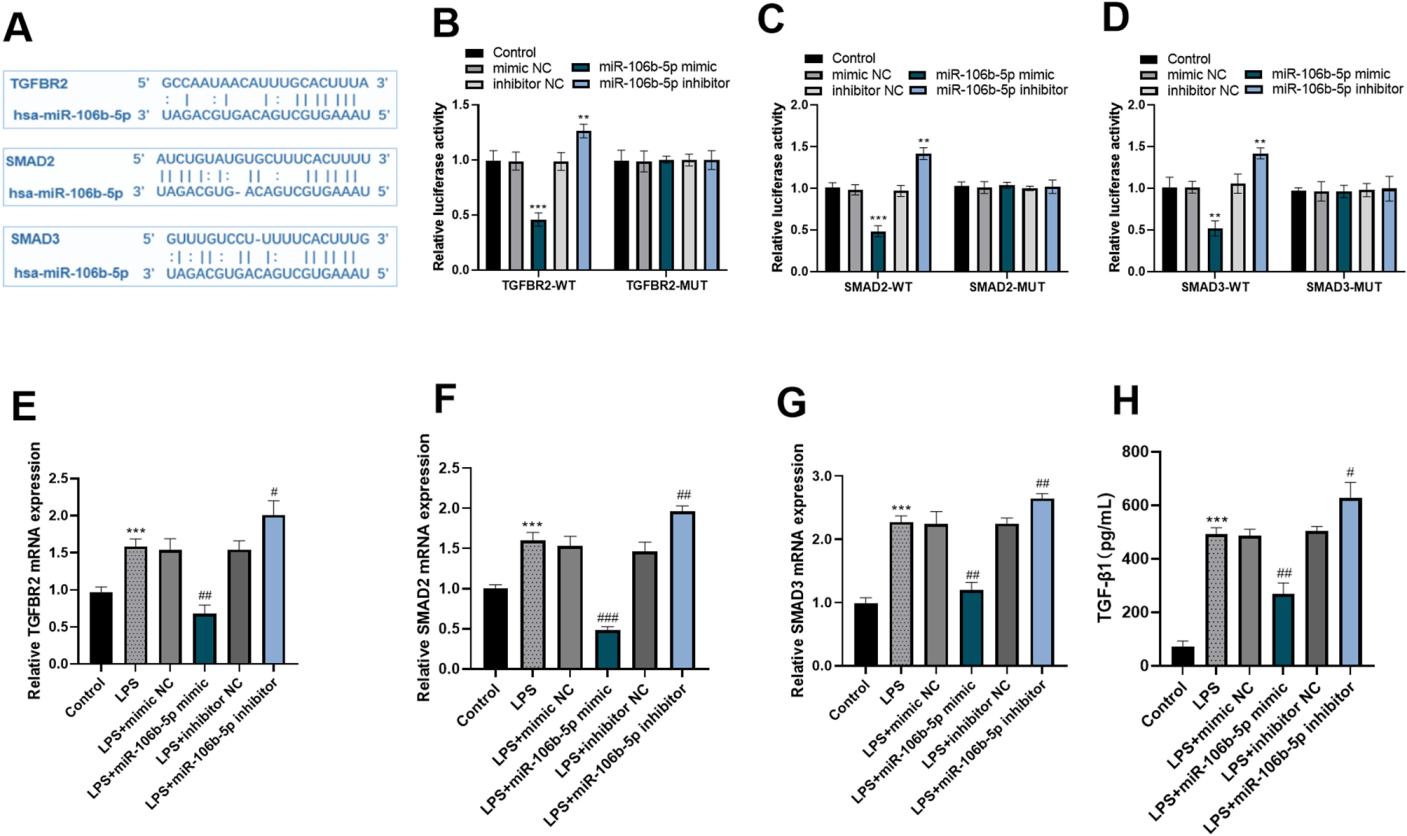
miR-106b − 5p targets and inhibits the TGF-β/SMAD pathway. The potential binding sites of miR-106b-5p with the targeted genes TGFBR2, SMAD2 and SMAD3 (**A**). Dual luciferase reporter gene assay was used to verify the targeted relationship (**B-D**). The overexpression of miR-106b-5p can inhibit the increase in the mRNA levels of TGFBR2, SMAD2 and SMAD3 induced by LPS (**E-G**). ELISA method was used to determine the effect of miR-106b-5p on the total secretion of TGF-β1 in the cell culture supernatant (**H**). * *p* < 0.05, ** *p* < 0.01, *** *p* < 0.001

Our findings suggest that miR-106b-5p acts as a protector of cellular function and demonstrates anti-inflammatory actions when it comes to the LPS-induced injury model of HK-2 cells. To elucidate the underlying mechanisms through which miR-106b-5p operates in LPS-treated cells, we conducted further investigations into its downstream signaling pathways. Through the analysis of online databases (https://rnasysu.com/encori/agoClipRNA.php), we were able to identify the potential target genes of miR-106b-5p. These genes were TGFBR2, SMAD2, and SMAD3, and we also determined their respective binding sites, as clearly depicted in Fig. 4A. To further validate the interactions between miR-106b-5p and these target genes, we conducted dual-luciferase reporter assays. The results of these assays confirmed the targeting relationships of miR-106b-5p with TGFBR2, SMAD2, and SMAD3 (Fig. 4B-D). Notably, LPS treatment led to a significant upregulation of TGFBR2, SMAD2, and SMAD3 in HK-2 cells, when compared to control conditions (*p*<0.01). Overexpression of miR-106b-5p effectively inhibited the increases in mRNA levels of TGFBR2, SMAD2, and SMAD3 induced by LPS. Conversely, silencing miR-106b-5p resulted in an elevation of these target expressions, thereby exacerbating the inflammatory effects of LPS (*p*<0.01) (Fig. 4E-G). ELISA results revealed that LPS treatment significantly elevated TGF-β1 levels compared to the control. Upregulating miR-106b-5p significantly suppressed TGF-β1, whereas miR-106b-5p knockdown had the opposite effect, indicating that miR-106b-5p inhibits TGF-β1 (*p*<0.05) (Fig. 4H).

## Discussion

MiRNAs have emerged as promising biomarkers in the progression of various human diseases, providing novel perspectives on the pathogenesis of CKD [18]. It has been demonstrated in recent studies that miR-27b-3p is of great significance in the inhibition of renal fibrosis [19]. Additionally, evidence indicates that miR-542-5p is implicated in the advancement of renal fibrosis [20]. In patients with advanced CKD, the levels of miR-223 in the serum are markedly lowered [21]. Our study also found miR-106b-5p downregulated in CKD. Previous and current findings suggest miR-27b-3p, miR-542-5p, miR-223 and miR-106b-5p as potential CKD diagnostic markers and therapeutic targets. Their interrelationships in CKD warrant further investigation, an intriguing research direction. Notably, our study shows miR-106b-5p expression declines with CKD progression, and its levels correlate positively with eGFR. These suggest miR-106b-5p regulates CKD development and may serve as an initial diagnostic biomarker. Building on prior work, this study advances clinical translation of CKD-related miRNAs and identifies miR-106b-5p as a new direction for early CKD management.

Furthermore, it should be noted that miR-106b-5p regulates cell proliferation, apoptosis, angiogenesis and inflammation in multiple physiological/pathological contexts, with its dysregulation linked to diabetes and hypertension. This study found no intergroup differences in diabetic/hypertensive patients and did not explore the relationship between comorbidities and miR-106b-5p levels. Future research will address this to inform miR-106b-5p-targeted precision therapies [22, 23]. Inflammation and oxidative stress have been established as critical factors contributing to the progression of CKD [24]. Renal inflammation serves as the primary catalyst for progressive renal damage, ultimately facilitating the onset and advancement of CKD [25]. Recent studies have highlighted the association of miR-146a with chronic kidney inflammation [26]. The results of our study indicate that in CKD patients, the levels of inflammatory biomarkers present in the serum rise gradually as the disease moves forward. Concurrently, miR-106b-5p demonstrates a significant negative correlation with these inflammatory mediators. However, the mechanisms through which miR-106b-5p exerts its immunomodulatory effects remain to be elucidated, warranting further experimental investigation. Furthermore, we further discovered that miR-106b-5p is downregulated by LPS in HK-2 cells, correlating with reduced viability, increased apoptosis, and inflammation. These results imply that miR-106b-5p has the potential to act as a therapeutic target for reducing LPS-induced kidney injury. It can achieve this by promoting cell survival and inhibiting the inflammatory response.

The TGF-β/Smad signaling pathway is a well-established mediator of inflammation, fibrosis, and apoptosis within the context of renal injury [27]. Specifically, the TGF-β1/Smad signaling cascade is intricately linked to the onset and progression of CKD [28]. Studies have shown that SMAD2 and SMAD3 are widely activated in renal fibrosis of CKD patients and animal models. These SMAD proteins are pivotal in the pathogenesis of kidney diseases and represent promising therapeutic targets; their inhibition may effectively mitigate the progression of CKD [29]. The initiation of TGF-β1 signaling occurs when it binds to the type II receptor of TGF-β (TGFBR2), which subsequently activates the downstream Smad2/3 pathway [30]. TGFBR2 is a fundamental component of the TGF-β signaling cascade and is critical to the processes of fibrosis, inflammation, and extracellular matrix (ECM) remodeling in CKD [31]. Our findings indicate that miR-106b-5p acts as a negative regulator in the TGF-β/Smad pathway, directly targeting TGFBR2 and its downstream effectors SMAD2 and SMAD3. By inhibiting the TGFBR2/SMAD2/3 pathway, miR-106b-5p reduces TGF-β1 levels. This regulatory mechanism suggests that upregulating miR-106b-5p may protect against renal injury by suppressing signals leading to inflammation and fibrosis. This study establishes the potential value of miR-106b-5p as a target for antifibrotic therapy in CKD.

Our research shows that the levels of miR-106b-5p in the serum of CKD patients are significantly reduced, and they are related to the development of the disease. However, the relatively limited sample size and the lack of long-term follow-up of CKD patients are the results of practical limitations encountered during the research. These factors underscore the necessity for further validation of the relationship between miR-106b-5p levels and CKD progression in larger cohorts. Additionally, our results demonstrate that miR-106b-5p remarkably lessens the LPS-induced injury, inflammatory processes, and apoptotic events in HK-2 cells. This indicates that miR-106b-5p might play a crucial part in the development of CKD, likely by regulating the TGF-β/Smad signaling pathway. Nevertheless, a more thorough and in-depth exploration is needed to fully understand the exact mechanisms behind these observed effects.

To conclude, miR-106b-5p is downregulated in CKD. It can inhibit LPS-induced damage in HK-2 cells, weaken the TGF-β/Smad pathway, and slow CKD progression. These research outcomes suggest that miR-106b-5p could potentially be a highly promising therapeutic target for addressing CKD, offering new hope for patients suffering from this condition (Graphical Abstract).

## Electronic supplementary material

Below is the link to the electronic supplementary material.

Supplementary Material 1

## Data Availability

The datasets used and/or analysed during the current study are available from the corresponding author on reasonable request.
