# Supplementary figures and images for: MiR-106b-5p improving the progression of chronic kidney disease by inhibiting the TGF-β/Smad pathway
Source: Hereditas. 2025 Jun 13;162:103. doi: 10.1186/s41065-025-00468-7 (PMC12164090; doi:10.1186/s41065-025-00468-7)

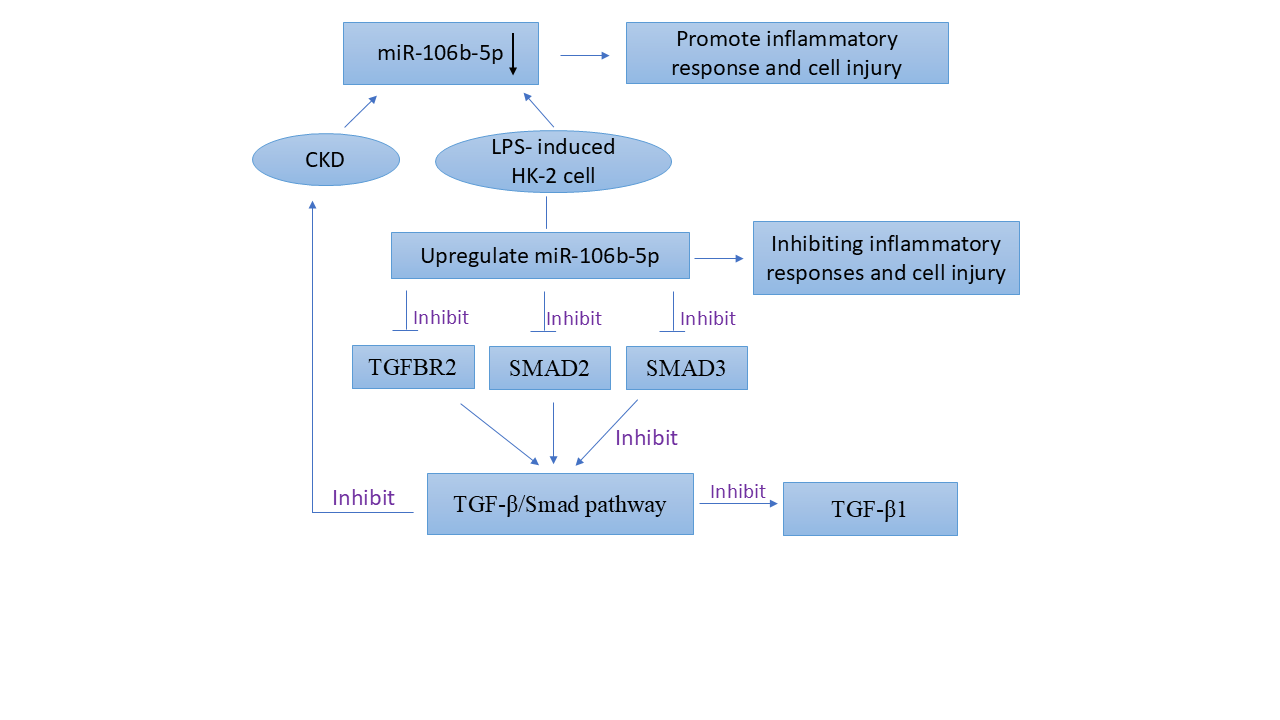

Supplement: Supplementary file 1 — Supplementary Material 1 [file 41065_2025_468_MOESM1_ESM.tif]
